# Central Composite Design Optimisation in Single Point Incremental Forming of Truncated Cones from Commercially Pure Titanium Grade 2 Sheet Metals

**DOI:** 10.3390/ma14133634

**Published:** 2021-06-29

**Authors:** Marcin Szpunar, Robert Ostrowski, Tomasz Trzepieciński, Ľuboš Kaščák

**Affiliations:** 1 Doctoral School of Engineering and Technical Sciences, Rzeszow University of Technology, al. Powst. Warszawy 12, 35-959 Rzeszów, Poland; d547@stud.prz.edu.pl; 2 Department of Materials Forming and Processing, Faculty of Mechanical Engineering and Aeronautics, Rzeszow University of Technology, al. Powst. Warszawy 8, 35-959 Rzeszów, Poland; rostrows@prz.edu.pl; 3 Institute of Technology and Material Engineering, Faculty of Mechanical Engineering, Technical University of Košice, Mäsiarska 74, 040 01 Košice, Slovakia; lubos.kascak@tuke.sk

**Keywords:** ANOVA, incremental sheet forming, sheet metals, single point incremental forming, SPIF

## Abstract

Single point incremental forming (SPIF) is an emerging process that is well-known to be suited for fabrication in small series production. The aim of this paper was to determine the optimal input parameters of the process in order to minimise the maximum of both the axial and the in-plane components of the forming force achieved during SPIF and the surface roughness of the internal surface of truncated-cone drawpieces. Grade 2 pure titanium sheets with a thickness of 0.4 mm were used as the test material. The central composite design and response surface method was used to determine the number of experiments required to study the responses through building a second-order quadratic model. Two directions of rotation of the forming tool were also considered. The input parameters were spindle speed, tool feed rate, and step size. The mathematical relations were defined using the response surfaces to predict the surface roughness of the drawpieces and the components of the forming force. It was found that feed rate has an insignificant role in both axial and in-plane forming forces, but step size is a major factor affecting axial and radial forming forces. However, step size directly affects the surface roughness on the inner surfaces of the drawpieces. Overall, the spindle speed −579 rpm (clockwise direction), tool feed 2000 mm/min, and step size 0.5 mm assure a minimisation of both force components and the surface roughness of drawpieces.

## 1. Introduction

Single point incremental forming (SPIF) is based on obtaining the desired shape of the drawpiece without special tooling [1]. A universal tool—a rotating pin which ends with a rounded tip—is used and this forms the desired shape from clamped sheet metal. The widespread use of CNC machines and robots in production enables SPIF to be applied in industry [2]. Incremental forming is justified in small batch production and enables the production of components that are impossible to form in a conventional deep-drawing process [3]. Compared with conventional sheet metal forming (SMF) methods, SPIF technology has many advantages, such as [4,5,6,7]:

- Sheet metal components with higher elongation can be made;
- Increased forming limits can be achieved;
- SPIF technology is more environmentally friendly than SMF methods;
- Surface quality and shape–dimensional accuracy are higher;
- As a die-less technology, SPIF does not require expensive stamping machines;
- It can replace SMF in small batch production.

In the SPIF process, many parameters that determine the accuracy and quality of the treated surface are controllable [8,9]. Among these parameters, the most important are: forming speed, step size, tool diameter, forming angle, rotational speed of the tool, tool shape, and coating [10,11]. Moreover, the choice of an appropriate lubricant depends on the deformation mechanisms that characterise the process [12,13,14]. The accuracy of SPIF-shaped drawpieces also depends on the anisotropy of the material’s mechanical properties and the springback phenomenon [15,16]. The issues related to the lubrication used and the methods of determining the coefficient of friction in SPIF have been discussed in a paper by Trzepieciński and Lemu [17].

It is possible to use this method for the production of titanium sheet products for the medical, automotive, and aviation industries. Ambrogio et al. [18] presented the use of SPIF for the production of a prosthesis using Grade 5 (Ti-6Al-4V) titanium alloy sheets. They proved that the process does not affect the biocompatibility of the products and enables the quick and cheap production of non-standard prostheses. Lu et al. [19] manufactured a cranial plate using the incremental sheet forming process with pure titanium sheets (Grade 1). They proved that this method has the potential for a real medical application—cranioplasty. Racz et al. [20] compared SPIF methods in the manufacture of cranioplasty plates from Grade 5 titanium alloy sheets, looking at different factors such as formability, microstructure, degree of control, roughness, energy consumption, accuracy, and production time, using the analytic hierarchy process. Peter et al. [21] applied ISF to create prototypes for automotive parts made of low carbon steel (DX54), aluminium alloy (EN AW-5083), and titanium (Grade 1 and Grade 5).

An adequate statistical method is required to properly understand the process. Response surface methodology (RSM) has recently become popular in analysing and optimising technological processes. Chauhan and Dass [22] applied RSM to investigate the dry sliding behaviour of Grade 5 titanium alloy. Rajesh and Varthanan [23] conducted experiments on the shot peening process in aluminium 2024-T3 using Ni shots to investigate fatigue strength, and then optimised the values of the process parameters using RSM. Bose and Nandi [24] developed a mathematical model of wire electrical discharge machining on a titanium hybrid composite using RSM and then determined the optimal solution. Veeraajay [25] used RSM to achieve the maximum wall angle and wall thickness with minimum surface roughness in the SPIF of Grade 5 titanium alloy. Saidi et al. [26] used RSM to determine the parameters of the SPIF process in order to minimise the maximum force achieved during forming of Grade 2 titanium sheets. Only tool diameter and step size were considered and the material flow used in the finite element (FE) model did not take into account the material anisotropy. Hashmi et al. [27] conducted FE-based simulations of the SPIF process of non-axisymmetric truncated pyramids with the aim of investigating the effect of both draw angle and tool/step size ratio, taking into account the anisotropic behaviour of sheets of Ti-6Al-4V titanium alloy. The analysis of thinning maps and shape errors highlighted that the tool/step size parameter plays a key role in SPIF. Mohanraj and Elangovan [28] performed experimental work and numerical analysis of Ti-6Al-4V incremental sheet forming, considering spindle speed, tool diameter, feed rate, and step size, to study the geometric accuracy and thinning of an aerospace component with complex shape. The simulation results show the applicability of the process in minimising production time in low-volume production. Maji and Kumar [29] developed RSM and adaptive neuro-fuzzy interference system models to predict a set of input parameters to achieve the desired output. It was found that the surface roughness was most significantly affected by step size. Lie et al. [30] applied the Taguchi-based optimisation method to optimise the process parameters for forming time in the SPIF of 7075-0 aluminium alloy. The most significant process parameters influencing forming time in the SPIF of truncated cones are the feed rate and the step size. Ali et al. [31] established the correlation between the maximum forming angle, the operating variables, and the surface roughness, using a gradient-boosting regression tree. The effect of the tool diameter and the feed rate on the maximum forming angle, surface topography, and microstructure was discussed.

In recent years, several numerical studies and pieces of experimental work were developed to optimise the SPIF of steel [32,33], commercially pure aluminium sheets [34], copper [35] and copper alloys [36], magnesium alloys [37], and aluminium alloy sheets [38,39]. Fewer studies were done using titanium sheets. Most of the research is focused on the minimisation of the surface finish of drawpieces. However, the optimisation of both the axial and the in-plane forces must also be widely considered in order to reduce the load on the machine tool and to manufacture eco-friendly products in low-volume production. Moreover, to the best of the authors’ knowledge, no study of the SPIF of titanium sheets has yet considered the direction of spindle rotation. As was found in preliminary studies, the direction of tool rotation with respect to the direction of tool movement has a crucial role in obtaining drawpieces of a specific height. In this paper, investigations of SPIF on Grade 2 titanium sheets were conducted using central composite design (CCD) with RSM. In the experiments, truncated cones were formed and the variable process parameters were spindle speed, tool feed, and step size. The aims of this paper were to find the values of process parameters in order to minimise the axial and radial components of the forming force achieved during the incremental forming process, and to optimise the surface roughness of the inner surface of the drawpieces while ensuring an appropriate height of the drawpiece.

## 2. Materials and Methods

### 2.1. Material

Commercially pure Grade 2 titanium sheets (Timet, Toronto, OH, USA) with a thickness of 0.4 mm were used as the test material. The chemical composition of the tested material delivered by Timet (Toronto, OH, USA) is listed in Table 1. The uniaxial tensile test was performed to determine the basic mechanical parameters of a titanium sheet at ambient temperature. The mechanical parameters are as follows: yield stress R_p0.2_ = 273 MPa, ultimate tensile stress σ_u_ = 359 MPa, strength coefficient K = 655 MPa, strain hardening exponent n = 0.137.

Titanium is a lightweight metal whose density is approximately 60% of that of steel. At service temperature, Grade 2 consists of 100% hexagonal close-packed α-phase. This material provides reasonable ductility and outstanding corrosion resistance in highly oxidising environments [40,41]. This combination of advantageous properties makes Grade 2 titanium a candidate for a large variety of aircraft, aerospace, and marine applications [42]. Some examples of aircraft and aerospace applications include ductwork, airframe skins, and brackets. It has also been widely used in chemical applications such as reaction vessels, cryogenic vessels, and condensers. In chemical and marine environments, it is used for tube headers in desalinisation plants and evaporation tanks.

### 2.2. Experimental Setup

**Figure 1:**
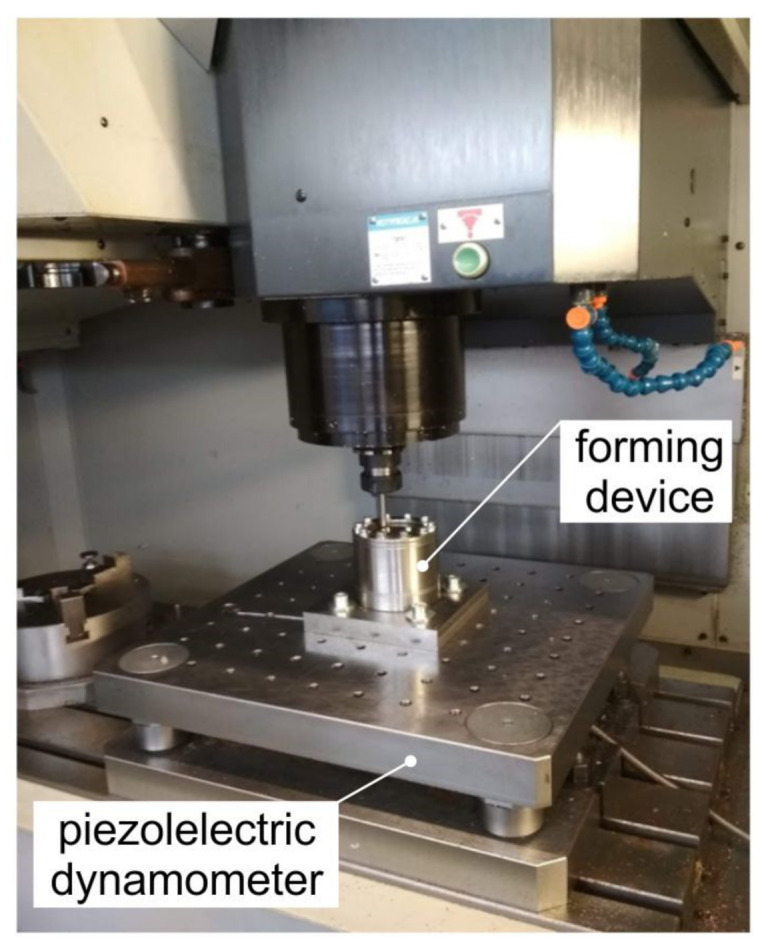
Test stand in the Makino PS95 vertical CNC milling machine.

**Figure 2:**
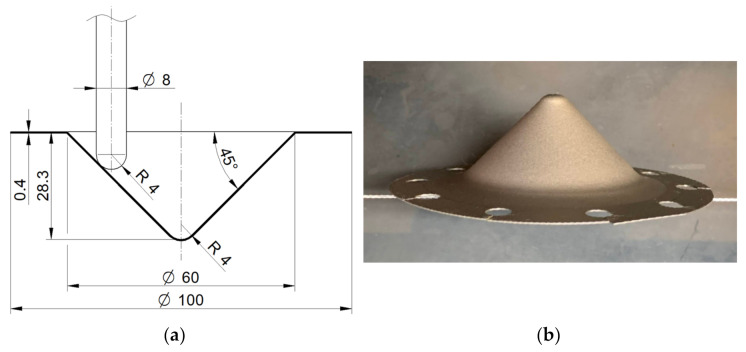
(**a**) Dimensions (in mm); and (**b**) view of a drawpiece obtained by incremental forming.

The investigations to form conical truncated drawpieces were carried out on a PS95 vertical CNC milling machine (Makino, Meguro, Japan). The experimental device, consisting of a body, a clamping plate, and press bolts, was mounted in a multi-component force plate of a piezoelectric dynamometer installed on a milling table (Figure 1). The specimens in the form of laser-cut circular blanks with a diameter of 100 mm were perfectly clamped in the device using bolts evenly arranged around the circumference. A tightening moment of 10 Nm was applied when tightening the bolts. A tungsten carbide tool with a diameter of 8 mm and a rounded tip with a radius of 4 mm was used. The geometrical parameters of the desired shape of the truncated cones (Figure 2a,b) were as follows: height 28.3 mm, slope angle 45° mm, and diameter of the base 60 mm. Fully synthetic 75W-85 lubricant (Castrol Ltd., Liverpool, UK) was used.

The axial (z-axis) and horizontal (x- and y-axes) forces occurring during the SPIF process were measured by a high-accuracy piezoelectric dynamometer (Kistler). It consisted of 4 force sensors calibrated in the range from 0 to 10 kN in the horizontal plane and from 0 to 60 kN in the vertical direction. The force values were acquired with a maximum sample rate per channel of 200 kHz. Based on the two horizontal components of forming force F_x_ and F_y_, the in-plane force F_xy_ was determined according to the following formula:

(1) $$F_{\mathrm{xy}}=\sqrt{F_{x}^{2}+F_{y}^{2}}$$

The aims of this paper were to determine the input parameters of the process in order to minimise the maximum axial and in-plane forces achieved during the incremental forming process, and to optimise the surface roughness of the inner surfaces of the drawpieces. Moreover, CCD was employed to determine the number of experiments required to study the responses through building a second-order quadratic model. The predominant input factors, which have most influence on the forming force and surface roughness during SPIF, were identified from previous work carried out. The input parameters were spindle speed n, tool feed rate f, and step size a_p_. The ranges of values of input parameters considered in the investigations are listed in Table 2. The CCD was composed of 5 levels, and 20 experiments (Table 3) were carried out to optimise the input variables. Design-Expert (Stat-Ease Inc., Minneapolis, MN, USA) software provided a prediction of equations in terms of actual units.

**Figure 3:**
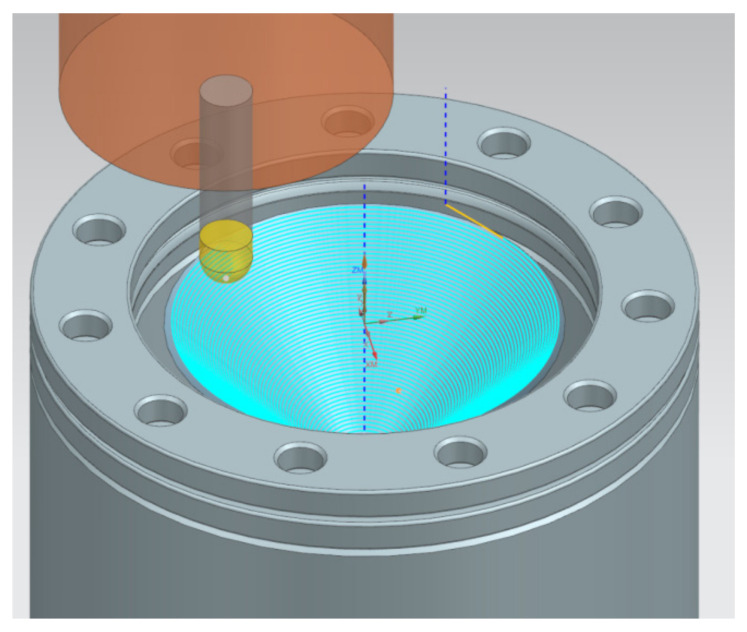
Tool path trajectory for a truncated cone.

The tool indents into the workpiece by step size and follows a spiral path for the desired part. The tool trajectory (Figure 3) was generated using NX CAM version 1938 software (Siemens, Munich, Germany) based on the numerical model of the desired shape of the drawpiece exported to the machining software.

**Figure 4:**
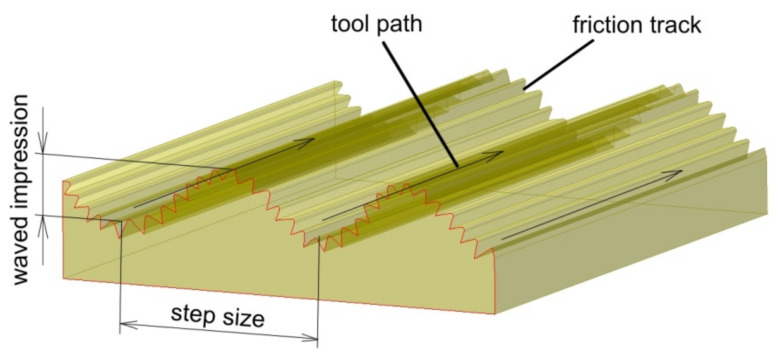
Surface of the drawpiece across the tool path.

The measurement of the surface roughness of the inner part of the drawpieces along the generating line of the cone was carried out using the MarSurf 400-series (Mahr, Göttingen, Germany). The 10-point peak–valley surface roughness Rz parameter was assumed to represent the roughness of the inner surface of the drawpieces after SPIF [43,44]. Hagan and Jeswiet [45] analysed the effect of step size on the surface roughness of incrementally formed drawpieces. It was found that due to the sinusoidal-type profile across the tool path (Figure 4), it is more useful to study the roughness of SPIFed parts using the Rz parameter. Moreover, Li et al. [44] selected the Rz parameter to describe the waved impression caused by the forming instability or the local bending of the workpiece.

## 3. Results and Discussion

### 3.1. Central Composite Design with RSM

When our central composite design experiments were carried out, the responses were analysed to obtain the optimal process parameters with regards to the surface roughness, the components of the forming forces, and the height of the drawpiece thus formed. To obtain significant factors that affect the SPIF of Grade 2 titanium sheets, analysis of variance (ANOVA) was performed. The purpose of the ANOVA was to study which process parameters significantly affect the Rz, both the axial and in-plane forces, and forming success. Fisher’s test was used to determine whether the specific parameter had a significant effect on the output parameter by comparing the F-test value with the table value (F_0.05_) at a significance level *α* = 0.05. The forming parameter is considered significant if the F-value is greater than F_0.05_. The aim of the modelling of both the forming and response parameters using RSM was to obtain the optimal response through second-order polynomial regression models.

Table 4 shows the results of the experimental tests based on the CCD. The values of the components of the forming forces correspond to the maximum force recorded during the forming of the drawpiece. Forming success h is determined from the possibility of obtaining a drawpiece with a tool height of 28.3 mm (Figure 2a) without the risk of cracking:

(2) $$h=\frac{28.3}{h_{t}}\cdot 100\%$$

where h_t_ is the tool cavity (drawpiece height) at the moment of crack formation; if a drawpiece without defects was obtained, then h_t_ = 28.3 mm.

Forming success h = 100% means that a drawpiece with a height of 28.3 mm (Figure 2a) was successfully formed.

**Figure 5:**
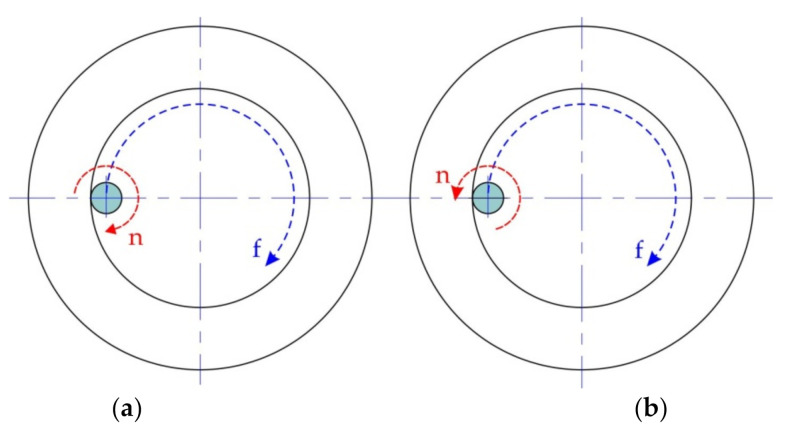
Schematic diagram of spindle rotation: (**a**) clockwise; and (**b**) anticlockwise.

**Figure 6:**
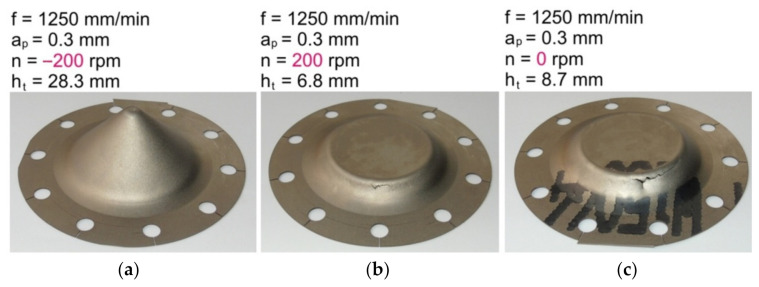
Drawpieces formed at a constant step size of 0.3 mm, tool feed rate of 1250 mm/min, and rotational speeds: (**a**) −200 rpm; (**b**) 200 rpm; and (**c**) 0 rpm (freely rotatable tool).

It was found that the direction of spindle rotation (Figure 5) significantly affects the formability of the Grade 2 titanium sheets, but only at a high speed of rotation of the spindle and at what is, concurrently, a small size of incremental step. Drawpieces formed with an incremental step size of 0.3 mm, maximum feed rate of 1250 mm/min, and spindle rate of −200 rpm (clockwise direction), were successfully formed (Figure 6a). However, drawpieces formed at 200 rpm (anticlockwise direction) and the same values of incremental step size and feed rate cracked at a height of 6.8 mm (Figure 6a). This is due to the fact that at 200 rpm the tool with the anticlockwise direction of rotation rolled on the surface of the sheet. As such, heating of the sheet was limited and the sheet metal cracked prematurely.

An additional test with a free rotatable tool with a step size of 0.3 mm and feed rate of 1250 mm/min led to cracking at a height of 8.7 mm (Figure 6c).

### 3.2. Axial Force F_z_

In order to analyse the effects of the input control factors on the in-plane force, a model of the response was developed. The effects of spindle speed, tool feed, and step size were analysed based on different plots. The adequacy of the response surface methodology models that were built was checked through ANOVA and the coefficient of determination R^2^. The equation, which is consistent with the experimental model and describes axial force (F_z_), is given in Equation (2) with the coded factors:

F_z_ = 586.135 + 0.619313A + 20.1055B + 86.5633C − 57.3425A^2^ − 16.887B^2^ − 30.2454C^2^ (3)

Table 5 presents the ANOVA results of the axial forming force at a confidence interval of 95%. To check the adequacy of the RSM model, the coefficients of determination of the responses R^2^ were found to be 0.9502. The regression model’s capability R^2^ was close to 0.9 indicating that the fitted regression model adequately aligned with the experimental data [46]. The predicted R^2^-value of 0.8672 was in reasonable agreement with the adjusted R^2^-value of 0.9273. The R^2^-values implied high mathematical validity of the regression Equation (2). From the ANOVA results for the responses, it was inferred that the model developed was adequate. The model F-value of 41.8 implied the model was significant.

In the significance testing of the null hypothesis, the *p*-value was the probability of obtaining test results at least as extreme as the results actually observed under the assumption that the null hypothesis was correct [47]. Statistically significant factors that affect the process were within the *p*-value range of 0–0.05. A *p*-value of 0.05–0.1 indicates marginally significant factors and a *p*-value above 0.1 indicates that the factor is insignificant in the process. It is clear that step size is a key parameter influencing the axial force, as also shown by Petek et al. [48]. Both the axial and the horizontal components of the forming force are directly related to the increase in step size [49]. The spindle speed has no direct influence on the forming force, but has a strong influence on the quality of the surface finish [48]. Parameters B, C, A^2^, and C^2^ are significant for the model; the R^2^ value obtained is 0.9502 for the axial force which means that the model is a 95.02% fit to predict the value of the response. Therefore, this model can be used to predict axial force in the design space. The step size is the factor having most influence on the value of the axial force in SPIF. The axial force also has a significant impact on the material formability due to excessive axial force tearing the sheet material [50]. It was also found by Murunden and Jung [46] that feed rate and step size favourably influence the response variable.

The lack of fit (LOF) test is used in the numerator in an F-test. The LOF F-value of 0.5137 (Table 5) implies the lack of fit is not significant relative to pure error. There is a 81.18% chance that a LOF F-value this large could occur due to noise.

**Figure 7:**
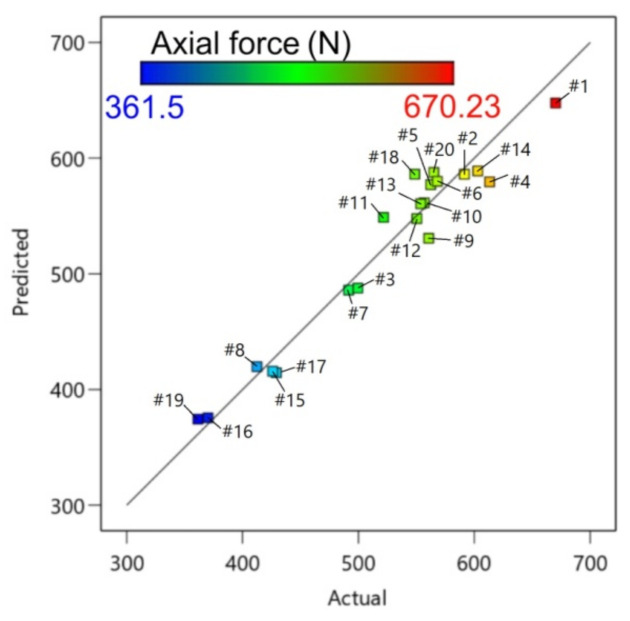
Predicted versus actual response for axial forming force.

**Figure 8:**
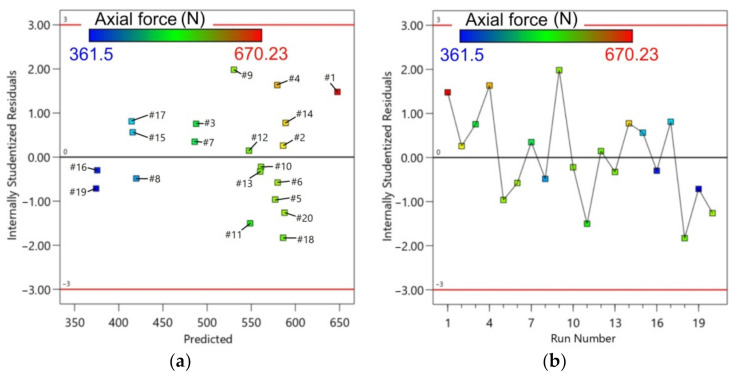
Residuals with reference to: (**a**) predicted; and (**b**) run number for axial forming force.

Figure 7 presents a plot for the axial force with predicted versus experimental values. The actual values were relatively close to the predicted straight line of regression. The proportional distribution of data around this line proves a good correlation between the predicted and experimental values. The residuals generally fall on a straight horizontal line (Figure 8), implying that the model errors are distributed normally [51]. The diagnostic analysis is supplemented by the normal probability plot of residuals also arranged along the straight line (Figure 8b).

**Figure 9:**
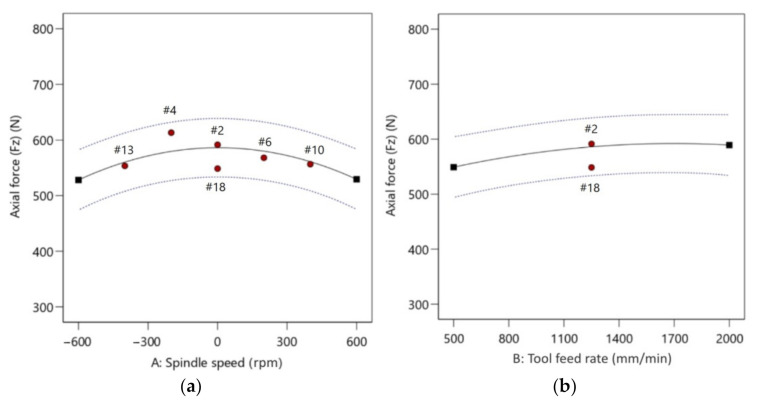

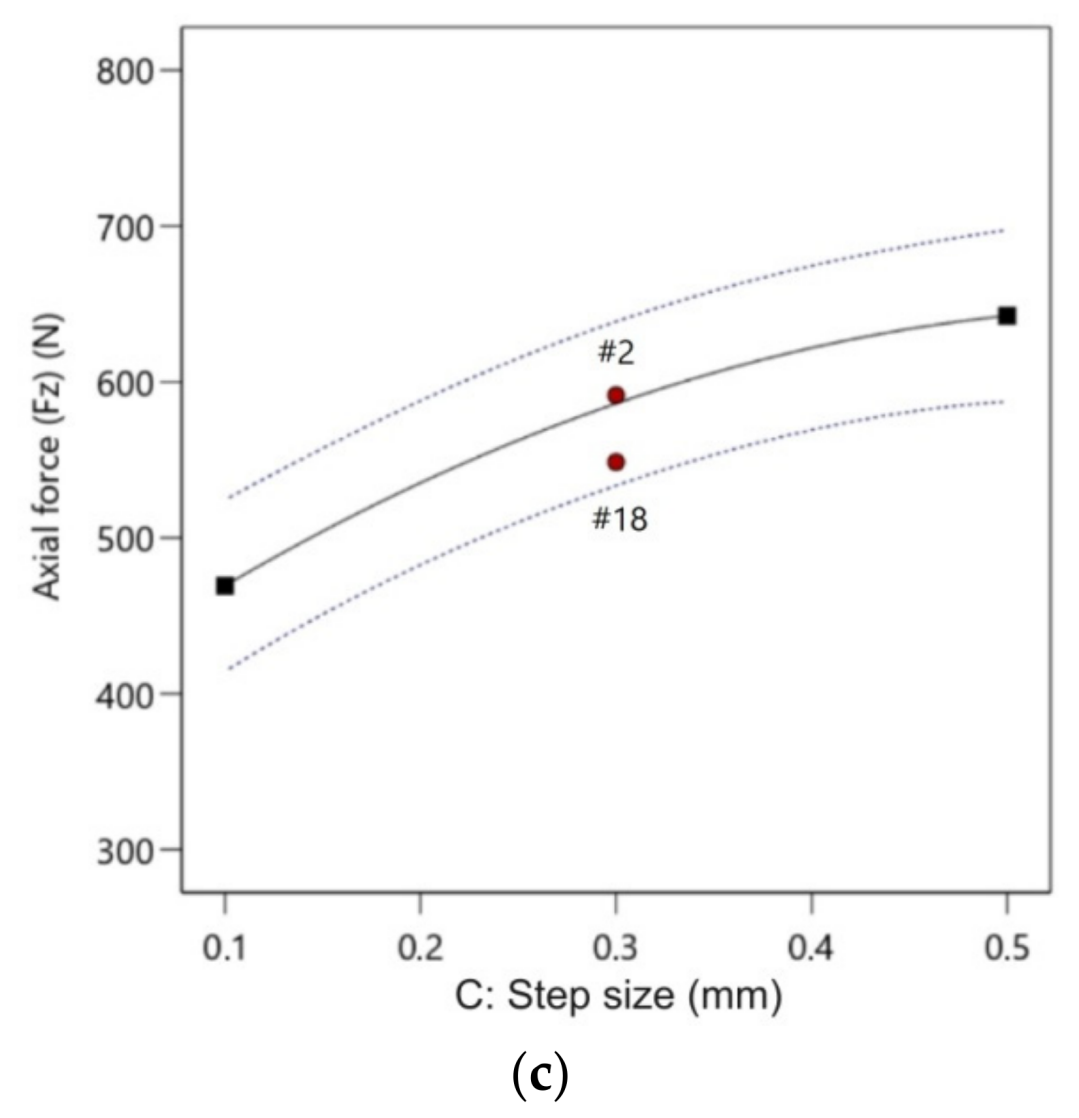
Plots showing the effect of: (**a**) spindle speed; (**b**) feed rate; and (**c**) step size on axial force.

One-factor plots explain the impact of individual factors and their effect on the axial force. From Figure 9, the following information can be obtained: In the range of the experiment, if spindle speed is higher, then axial force is lower, 590 N for 0 rpm and 528 N for 600 rpm—the temperature achieved by friction plays a key role in reducing the axial forming force (Figure 9a). With an increase in feed rate from 500 to 2000 mm/min there was a slight change in the axial forming force from 549 N to 589 N (Figure 9b). This indicates that feed rate has an insignificant role in the axial forming force. With an increase in step size from 0.1 mm to 0.5 mm, the axial force increased from 469 N to 642 N, indicating that step size is a major factor affecting the axial forming force (Figure 9c).

### 3.3. In-Plane Force F_xy_

**Figure 10:**
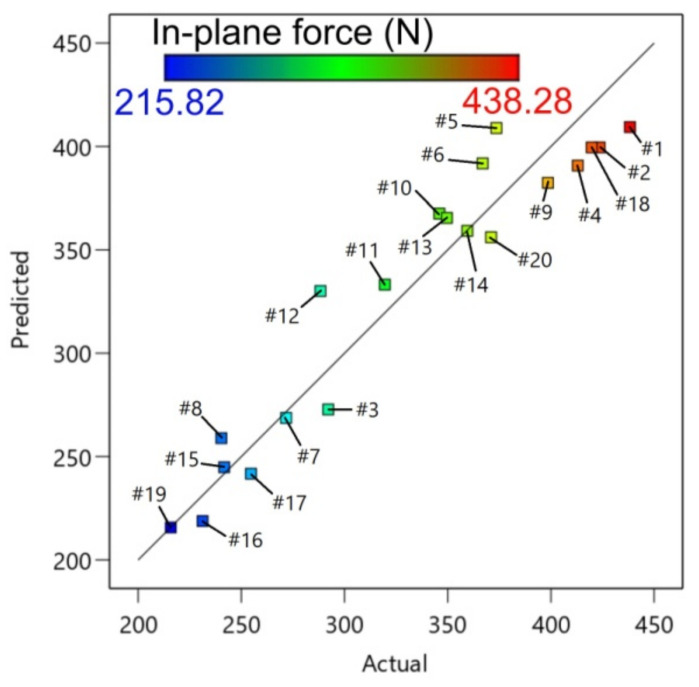
Predicted versus actual response for in-plane force.

**Figure 11:**
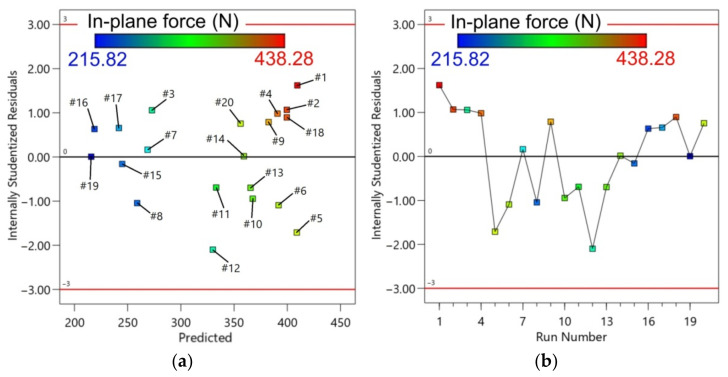
Residuals with reference to: (**a**) predicted; and (**b**) run number for in-plane force.

Table 6 presents the ANOVA for the in-plane force at a 95% confidence interval. Model terms that were significant were C, A^2^, B^2^, and, marginally, B. The value of R^2^ achieved was 0.9142, which means that the model is 91.42% capable of predicting the response. The R^2^-value was in reasonable agreement with the adjusted R^2^ (0.8836) and the predicted R^2^ (0.8159). Therefore, the model as designed can be applied to predict the in-plane force in the selected ranges of the parameter. The F-value of 29.83 implies significance of the model. Figure 10 presents the plot of the predicted against the experimental values of the in-plane force. As illustrated in Figure 11, the regression model tends to have error randomness without too many outliers, and a normal distribution in terms of residuals. The equation with a coded factor that fits the experimental model is given in Equation (4):

F_xy_ = 399.528 + 1.55562A + 13.0144B + 57.1742C − 74.3351A^2^ − 37.7303C^2^ (4)

**Figure 12:**
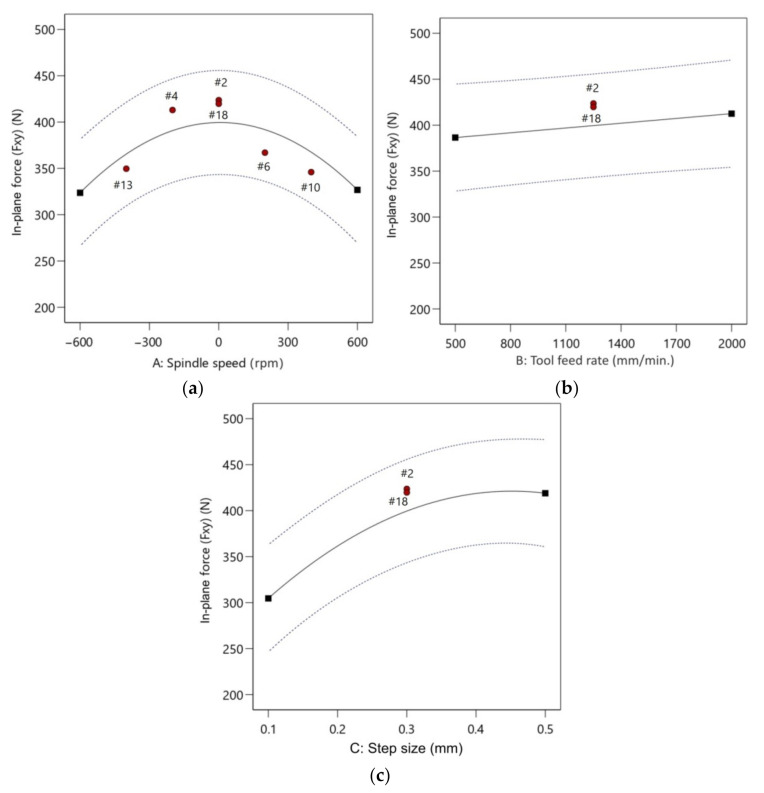
Plots showing the effect of: (**a**) spindle speed; (**b**) feed rate; and (**c**) step size on in-plane force.

The analysis of one-factor plots explains the impact of individual factors on the radial forming force. In the range of the experiment, higher spindle speed produces a lower in-plane force, 400 N for 0 rpm and 324 N for 600 rpm, which means that the temperature produced by friction is a significant factor in the reduction of the in-plane force (Figure 12a). With an increase in the feed rate from 500 to 2000 mm/min, there is a slight change in the in-plane force from 387 N to 413 N (Figure 12b). This indicates that the feed rate does not play a key role in the in-plane force. Similar observations were found by Özgen et al. [52]. As the step size increases from 0.1 mm to 0.5 mm, the in-plane force changes from 305 N to 421 N. This indicates that the step size is a major parameter affecting the in-plane force (Figure 12c).

### 3.4. Surface Roughness Parameter Rz

The results of ANOVA for the surface roughness parameter are presented in Table 7. The significant terms of the model are C, AB, BC, A^2^, and C^2^; A is marginally significant (non-significant). This conclusion is also consistent with the results of the Box–Behnken design analysed by Yao et al. [53]. The surface roughness was influenced most by the step down. The most important parameter which influences the surface roughness is step size due to it being characterised as resulting from large-scale waviness created by the path of the forming tool [54,55,56]. It was also found by Shanmuganatan and Kumar [57] that the significance of the step size is the most predominant for the response of surface roughness. The value of R^2^ obtained was 0.9077, which means that the model is 90.77% able to predict the response. The adjusted R^2^-value (0.8539) and predicted R^2^-value (0.8009) were in reasonable agreement. For this reason, the model as determined can be used to predict the parameter Rz in the parameter ranges used in the experiments. The regression equation which fits the experimental data is given in Equation (5):

Rz = 9.18172 − 0.36435A − 0.103397B − 1.12494C − 0.973022AB − 1.45625BC + 1.86651A^2^ + 0.837295C^2^ (5)

**Figure 13:**
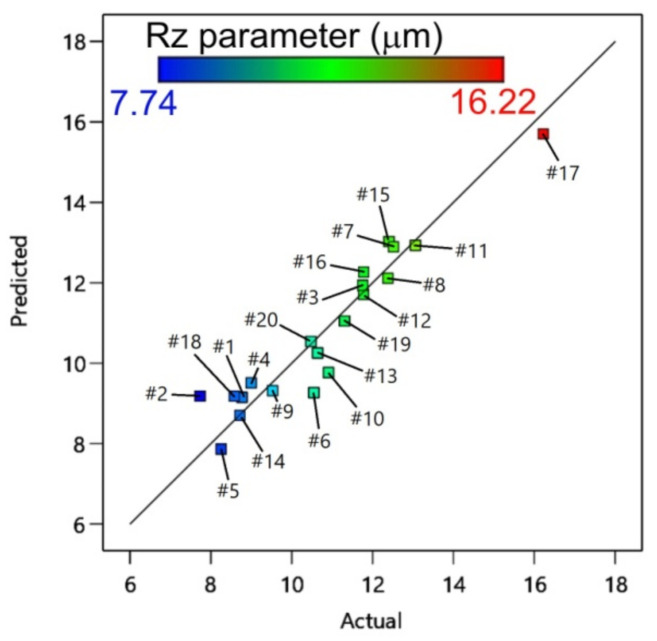
Predicted versus actual response for the surface parameter Rz.

**Figure 14:**
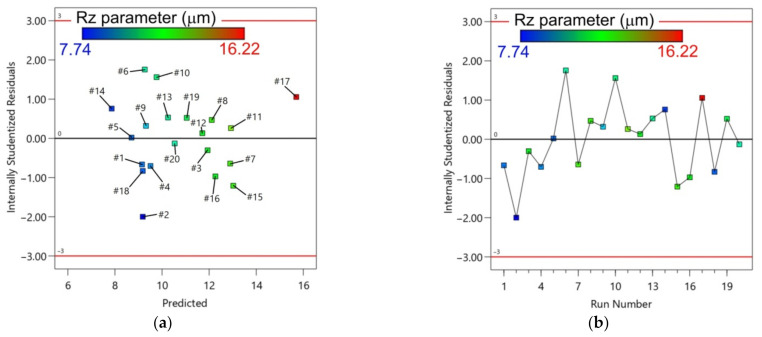
Residuals with reference to: (**a**) predicted; and (**b**) run number for the surface parameter Rz.

Figure 13 shows a strong correlation between the actual and predicted values of the surface roughness parameter Rz. The distribution of the residuals is shown using a plot between the predicted and residual responses (Figure 14). The points between boundary lines show no definite structure [58,59].

**Figure 15:**
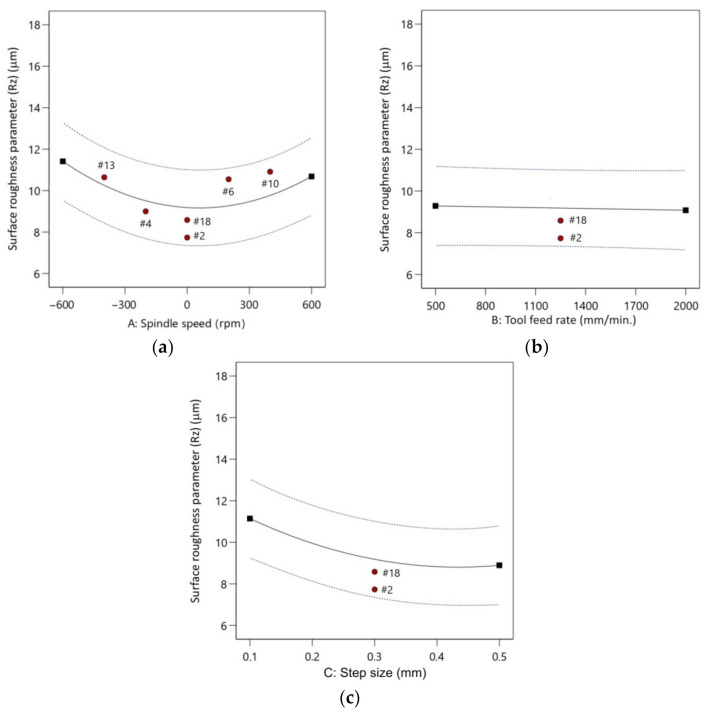
The plots showing the effect of: (**a**) spindle speed; (**b**) feed rate; and (**c**) step size on the parameter Rz.

As the spindle speed increases, the parameter Rz worsens from 8.581 µm to 11.412 µm (Figure 15a). This means that the friction affects the surface quality [60,61]. The results are consistent with the findings of an investigation conducted by Bagudanch et al. [62]. They found that increasing the spindle speed strongly affected the deterioration of the surface finish of a workpiece. In the range of the experiment, the tool feed rate has no direct effect on parameter Rz (Figure 15b). When the feed rate changes from 500 mm/min to 2000 mm/min, the surface parameter Rz oscillates between 8.581 µm and 9.285 µm. The step size produces a significant variation in surface roughness from Rz 11.144 µm for 0.1 mm to Rz 8.894 µm for 0.5 mm (Figure 15c).

### 3.5. Forming Success h

**Figure 16:**
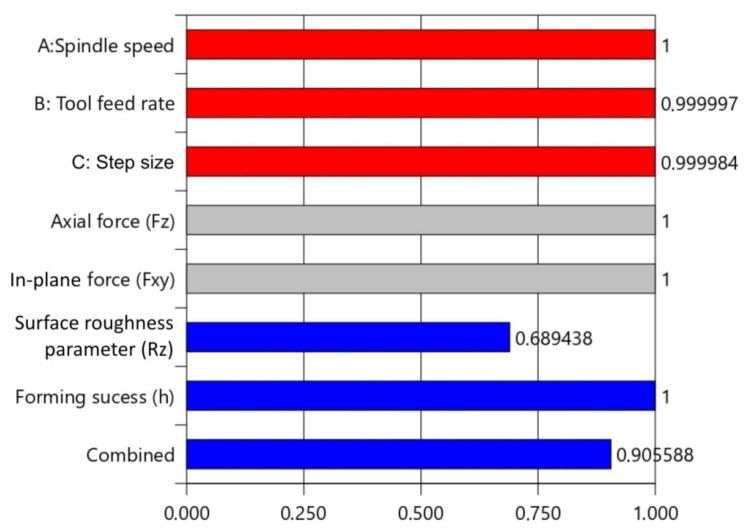
Desirability plot of the most favourable solution.

Desirability-based optimisation was performed based on the desirability of multi-response. Desirability D = 0 indicates that a response is unacceptable, while D = 1 indicates that a response accurately meets the target value. Table 8 presents the constraints for the optimisation of the surface roughness parameter Rz and the forming success index h. As an optimal value, the solution with the highest desirability value is chosen. The input factors are represented by the red histogram (Figure 16). Responses (Rz and h) are represented by blue bars. The bottom blue bar shows the desirability of all responses and factors.

**Figure 17:**
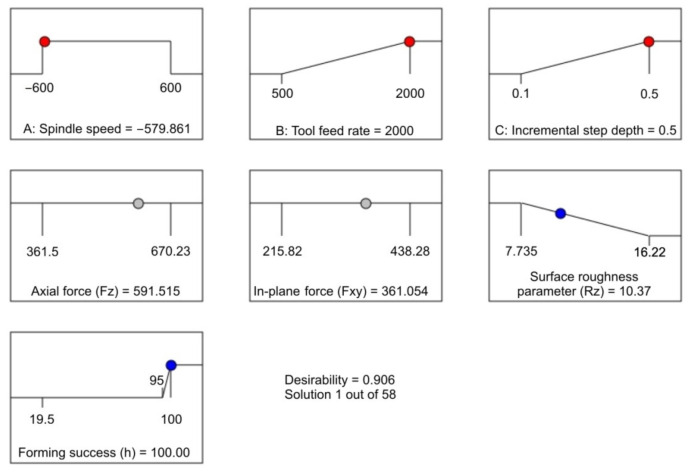
Ramp plot of optimisation solution.

The ramp plot in Figure 17 shows the desirability for each factor in each response, as well as the combined desirability. The higher up the ramp, the better the desirability [63]. Output responses such as the surface roughness parameter Rz and the forming success are taken into account. The best combination of solutions is Rz = 10.370 µm and h = 100%, which can be achieved when forming with a spindle speed of 580 rpm, feed rate 2000 mm/min, and step size 0.5 mm (Figure 17). The predicted axial forming force and in-plane force at the optimal forming parameters are F_z_ = 591.52 N and F_xy_ = 361.06 N, respectively. The desirability for the optimum parameters was 90.6% (Table 8).

### 3.6. Validation Run

A validation run was carried out using the parameters obtained from the desirability-based optimisation (Table 9). The confirmation run produced an element with a surface roughness parameter Rz = 10.14 µm with forming forces measured as: axial force F_z_ = 582.44 N, in-plane force F_xy_ = 375.61, and without sheet cracking (h = 100%). The values achieved for surface roughness and both the axial and in-plane forces deviate by 2.2%, 1.6%, and 4%, respectively.

## 4. Conclusions

This paper investigated the process parameters influencing the surface roughness and components of the forming force in the SPIF of Grade 2 titanium sheets. The response surface methodology together with ANOVA was utilised to determine the best combination of forming parameters to minimise the forming force and surface roughness. The following conclusions can be drawn from the results:

- The direction of tool rotation in relation to the feed direction is one of the key SPIF parameters influencing the possibility of receiving a Grade 2 titanium drawpiece without the risk of cracking. Drawpieces formed with clockwise tool rotation exhibit higher height without the risk of cracking;
- The direction of spindle rotation significantly affects the formability of Grade 2 titanium sheets, but only at a high speed of rotation of the spindle with an accompanying small step size;
- By increasing spindle speed, a reduction in forming forces was observed;
- Samples formed with high values of spindle speed showed poor surface qualities;
- A major factor affecting forming forces is step size;
- The archived R^2^ value equals 0.9502, 0.9142, and 0.9077 for the axial forming force, the in-plane forming force, and the surface roughness parameter Rz, respectively, signifying that the second-order polynomial regression models are 95.02%, 91.42%, and 90.77% able to predict the response value;
- The optimal forming parameters minimising the surface roughness and axial and in-plane components of the forming force are as follows: spindle speed −580 rpm, feed rate 2000 mm/min, and step size 0.5 mm;
- The experiments performed with the optimal parameters produced a 2.2%, 1.6%, and 4% discrepancy with the model as regards surface roughness, axial force, and in-plane force, respectively.

## Figures and Tables

**Table 1:** Chemical composition of the Grade 2 titanium sheet (in wt.%).

Fe	C	O	N	Ti
0.12	0.009	0.23	0.009	balance

**Table 2:** Factors and levels used in the CCD.

Forming Parameter	Factor	Unit	Low Level	High Level
Spindle speed n	A	rpm	−600	600
Tool feed rate f	B	mm/min	500	2000
Step size a_p_	C	mm	0.1	0.5

**Table 3:** Plan of experiments for CCD optimisation.

Number of Experiment	Spindle Speed, rpm	Feed Rate, mm/min	Step Size, mm
1	0	1250	0.563215
2	0	1250	0.3
3	789.644	1250	0.3
4	−200	1250	0.3
5	200	2237.06	0.3
6	200	1250	0.3
7	−789.644	1250	0.3
8	0	1250	0.0367852
9	0	262.944	0.3
10	400	1250	0.3
11	600	500	0.5
12	−600	500	0.5
13	−400	1250	0.3
14	600	2000	0.5
15	600	2000	0.1
16	600	500	0.1
17	−600	2000	0.1
18	0	1250	0.3
19	−600	500	0.1
20	−600	2000	0.5

**Table 4:** Results of the SPIF forming.

Std.	Run	A: Spindle Speed, rpm	B: Feed Rate, mm/min	C: Step Size, mm	Axial Force F_z_, N	In-Plane Force F_xy_, N	Surface Parameter Rz, µm	Forming Success h, %
14	1	0	1250	0.563215	670.23	438.28	8.772	27
15	2	0	1250	0.3	591.49	423.64	7.735	23.4
10	3	789.644	1250	0.3	499.63	292.15	11.757	100
19	4	−200	1250	0.3	613.24	413.02	8.999	100
12	5	200	2237.06	0.3	562.4	373.59	8.718	27.9
16	6	200	1250	0.3	568.08	366.95	10.547	24
9	7	−789.644	1250	0.3	491.62	271.71	12.518	100
13	8	0	1250	0.0367852	412.44	240.35	12.38	19.5
11	9	0	262.944	0.3	560.38	398.58	9.528	19.8
17	10	400	1250	0.3	556.51	345.96	10.91	100
6	11	600	500	0.5	521.73	319.57	13.06	100
5	12	−600	500	0.5	550.16	288.34	11.777	100
18	13	−400	1250	0.3	553.5	349.6	10.641	100
8	14	600	2000	0.5	603.06	359.52	8.253	100
4	15	600	2000	0.1	426.07	241.7	12.407	30.6
2	16	600	500	0.1	370.25	231.27	11.779	100
3	17	−600	2000	0.1	429.13	254.68	16.22	100
20	18	0	1250	0.3	548.59	419.76	8.581	20.9
1	19	−600	500	0.1	361.5	215.82	11.307	100
7	20	−600	2000	0.5	565.1	371	10.475	100

**Table 5:** ANOVA results of the axial force in SPIF.

Source	Sum of Squares	Degrees of Freedom	Mean Square	F-Value	*p*-Value	Significance
Model	1.259 × 10^5^	6	20,984.00	41.38	<0.0001	significant
A-Spindle speed	4.84	1	4.84	0.0095	0.9236	–
B-Feed rate	4626.49	1	4626.49	9.12	0.0098	–
C-Step size	85,902.91	1	85,902.91	169.38	<0.0001	–
A^2^	20,462.71	1	20,462.71	40.35	<0.0001	–
B^2^	2008.14	1	2008.14	3.96	0.0681	–
C^2^	6495.64	1	6495.64	12.81	0.0034	–
Residual	6592.93	13	507.15	–	–	–
LOF	5672.72	12	472.73	0.5137	0.8118	not significant
Pure Error	920.20	1	920.20	–	–	–
Cor Total	1.325 × 10^5^	19	–	–	–	–
Std. Dev.	22.52	–	–	–	–	–
Mean	522.76	–	–	–	–	–
C.V. %	4.31	–	–	–	–	–

**Table 6:** ANOVA results of the in-plane force in SPIF.

Source	Sum of Squares	Degrees of Freedom	Mean Square	F-Value	*p*-Value	Significance
Model	89,185.94	5	17,837.19	29.83	<0.0001	significant
A-Spindle speed	30.62	1	30.62	0.0512	0.8242	–
B-Feed rate	1938.55	1	1938.55	3.24	0.0933	–
C-Step size	37,474.84	1	37,474.84	62.68	<0.0001	–
A^2^	34,865.34	1	34,865.34	58.31	<0.0001	–
C^2^	10,453.82	1	10,453.82	17.48	0.0009	–
Residual	8370.66	14	597.90	–	–	–
LOF	8363.13	13	643.32	85.47	0.0845	not significant
Pure Error	7.53	1	7.53	–	–	–
Cor. Total	97,556.60	19	–	–	–	–
Std. Dev.	24.45	–	–	–	–	–
Mean	330.77	–	–	–	–	–
C.V. %	7.39	–	–	–	–	–

**Table 7:** ANOVA results of the surface roughness parameter Rz in SPIF.

Source	Sum of Squares	Degrees of Freedom	Mean Square	F-Value	*p*-Value	Significance
Model	72.54	7	10.36	16.87	<0.0001	significant
A-Spindle speed	1.68	1	1.68	2.73	0.1241	–
B-Tool feed	0.1219	1	0.1219	0.1985	0.6639	–
C-Step size	14.51	1	14.51	23.61	0.0004	–
AB	7.70	1	7.70	12.54	0.0041	–
BC	16.97	1	16.97	27.61	0.0002	–
A^2^	21.96	1	21.96	35.75	<0.0001	–
C^2^	5.14	1	5.14	8.37	0.0135	–
Residual	7.37	12	0.6144	–	–	–
LOF	7.01	11	0.6377	1.78	0.5305	not significant
Pure Error	0.3579	1	0.3579	–	–	–
Cor. Total	79.92	19	–	–	–	–
Std. Dev.	0.7838	–	–	–	–	–
Mean	10.82	–	–	–	–	–
C.V. %	7.25	–	–	–	–	–

**Table 8:** Limits used and goals for optimisation.

Constraints Name	Goal	Lower Limit	Upper Limit
A: Spindle speed	is in range	−600	600
B: Feed rate	maximise	500	2000
C: Step size	maximise	0.1	0.5
Axial force (F_z_)	none	361.5	670.23
In-plane force (F_xy_)	none	215.82	438.28
Surface roughness parameter (Rz)	minimise	7.735	16.22
Forming success (h)	maximise	95	100

**Table 9:** The most favourable optimal global solution.

Spindle Speed, rpm	Tool Feed, mm/min	Step Size, mm	Axial Force F_z_, N	In-Plane Force F_xy_, N	Surface Roughness Parameter Rz, μm	Forming Success (h)	Desirability D
−579.844	2000	0.500	591.518	361.058	10.370	100.00	0.906

## Data Availability

The data presented in this study are available on request from the corresponding author.
